# Beyond resistance: social factors in the general public response to pandemic influenza

**DOI:** 10.1186/s12889-015-1756-8

**Published:** 2015-04-29

**Authors:** Mark D M Davis, Niamh Stephenson, Davina Lohm, Emily Waller, Paul Flowers

**Affiliations:** School of Social Sciences, Monash University, Melbourne, Victoria Australia; School of Public Health and Community Medicine, University of New South Wales, Sydney, Australia; School of Health and Life Sciences, Glasgow Caledonian University, Glasgow, Scotland

## Abstract

**Background:**

Influencing the general public response to pandemics is a public health priority. There is a prevailing view, however, that the general public is resistant to communications on pandemic influenza and that behavioural responses to the 2009/10 H1N1 pandemic were not sufficient. Using qualitative methods, this paper investigates how members of the general public respond to pandemic influenza and the hygiene, social isolation and other measures proposed by public health. Going beyond the commonly deployed notion that the general public is resistant to public health communications, this paper examines how health individualism, gender and real world constraints enable and limit individual action.

**Methods:**

In-depth interviews (n = 57) and focus groups (ten focus groups; 59 individuals) were conducted with community samples in Melbourne, Sydney and Glasgow. Participants were selected according to maximum variation sampling using purposive criteria, including: 1) pregnancy in 2009/2010; 2) chronic illness; 3) aged 70 years and over; 4) no disclosed health problems. Verbatim transcripts were subjected to inductive, thematic analysis.

**Results:**

Respondents did not express resistance to public health communications, but gave insight into how they interpreted and implemented guidance. An individualistic approach to pandemic risk predominated. The uptake of hygiene, social isolation and vaccine strategies was constrained by seeing oneself ‘at risk’ but not ‘a risk’ to others. Gender norms shape how members of the general public enact hygiene and social isolation. Other challenges pertained to over-reliance on perceived remoteness from risk, expectation of recovery from infection and practical constraints on the uptake of vaccination.

**Conclusions:**

Overall, respondents were engaged with public health advice regarding pandemic influenza, indicating that the idea of public resistance has limited explanatory power. Public communications are endorsed, but challenges persist. Individualistic approaches to pandemic risk inhibit acting for the benefit of others and may deepen divisions in the community according to health status. Public communications on pandemics are mediated by gender norms that may overburden women and limit the action of men. Social research on the public response to pandemics needs to focus on the social structures and real world settings and relationships that shape the action of individuals.

## Background

The re-emergence of infectious diseases is a leading public health problem. Pandemics and epidemics [1] – including Avian influenza, SARS, Ebola, and pandemic influenza – and the rise of anti-microbial organisms [2] now threaten the health of populations around the globe. It has been argued that the re-emergence of these diseases marks the end of the golden age of medicine and the dawning of a period where health and security will be undermined by resurgent infectious diseases [3]. Pandemic influenza stands out in this situation because: it spreads quickly around the globe affecting many millions of people; it is associated with, potentially, high mortality, and; the world experienced a highly publicised, though ultimately mild for most, pandemic influenza in 2009/10. It is believed that another, more serious influenza pandemic is inevitable, though no-one, as yet, can predict when it will occur.

For these reasons, explaining infectious diseases threats to the general public and encouraging them to adapt their health behaviours is high on the public health agenda. In relation to pandemic influenza, public communications feature in preparedness and response planning which requires that members of the general public adopt measures during a public health emergency, including: hygiene (e.g., covering the mouth and nose when sneezing or coughing, washing hands, keeping surfaces clean, avoiding sharing personal items) and the avoidance of close contact with others [4]. Understanding how populations respond is also crucial for the science that supports response planning. For example, mathematical models, which underpin pandemic response planning, factor in biological, psychological and sociological assumptions of how populations respond to infectious diseases [5,6]. Effective communications with the general public and understanding how they respond, therefore, have a pivotal role to play in the management of pandemic influenza, in particular, and in the area of emerging infectious diseases, in general.

However, knowledge of how to best communicate on pandemics with the general public and how they take up these messages is an emerging field with some inconsistencies [7]. Evaluations of the public health response to the 2009/10 pandemic influenza claim that public communications were largely successful in preparing and reassuring publics during the emergency [8,9]. These findings need to be read against the fact that the pandemic was a short-lived and ultimately mild public health emergency for most people.

There is a view, also, that members of the general public are resistant to pandemic risk messages. Some commentary has suggested that the general population is increasingly resistant to public policy on global threats, including climate change and emerging infectious diseases [10]. Surveys – which dominate the social scientific view on public responses – conducted during the 2009 pandemic indicate that populations in the UK and Australia were complacent with regard to H1N1 and reported insufficient behavioural responses [11-15]. Broad brush, risk communication research has identified that material circumstances and symbolic framing of risk [7], inequalities in education and access to media [16], (mis)trust in media and governmental advice [17,18], all shape how members of the general public respond to communications on pandemics. Close-focus, qualitative research offers the view that while the general public endorses governmental advice, in the circumstances of the 2009/10 pandemic they were also unlikely to act in the ways advised by governments [19,20].

There are additional explanations for the apparent resistance on the part of the general public. For example, because they are bombarded with so many messages, including those pertaining to pandemics, members of the general public may by subject to ‘health threat fatigue’ [21]. This is not the same as resistance. It is, instead, a dulling of alertness seated in screening out of overwhelming and competing risk messages. Members of the general public appear to digest and critically reflect on risk communications messages [22], and tailor risk reduction strategies to their personal circumstances [23]. It is also argued that the general public is only too aware of the ‘boy who cried wolf’ syndrome [24], where too frequent assertion of danger leads publics to dismiss public health warnings. In addition, audience reception of communications on health is framed by the historic rise of individualism in society [25] and health systems [26]. Individualism implies that members of the general public take on the view that responsibility for their health is a matter of personal volition and effort. This view is often utilised in health communications that call on people to take care of themselves, but it is a perspective that can obscure factors that are not within the control of the individual. It is also an approach to risk that has a moral loading and therefore a negative effect for those who are unable – through choice or otherwise – to avoid health harms. Exactly how individualism plays out in relation to pandemic influenza warrants further inquiry.

Because it is so vital that public health authorities communicate with members of the general public as effectively as possible and as there are competing explanations and routes of inquiry available in the literature, it is necessary to re-examine the apparent resistance to communications and advice on the part of the general public. A central challenge is to get beyond prevailing assumptions and build up a theory of public engagement informed by the life worlds of the general public [7]. Understanding why populations fail to sufficiently enact precautions must involve taking account of how lives are lived and the meanings ascribed to the threat of infectious diseases. Indeed, what might look like lack of precaution may turn out to be reasonable given the material and symbolic circumstances of affected individuals and populations. A related challenge, then, is re-examining how public health characterises the general public in research on pandemics and in the more general area of emerging infectious diseases. Taking these steps is vital to ensure that the public health response and its communications with the general public are as resonant, meaningful and effective as possible.

This paper, therefore, uses inductive, qualitative research methods to develop new knowledge on how members of the general population respond to pandemic influenza, set against the backdrop of the assumed resistance on the part of the general public and related critiques, including, health risk fatigue, the risk communication dilemma and individualism. The analysis poses the question:

How do members of the general public respond to the threat of pandemic influenza and to the hygiene, social isolation and other measures proposed by public health?

By addressing this question in the manner indicated, the paper offers an alternative framing of pandemic influenza perceptions and behaviours in an effort to contribute to the better health of individuals and populations facing risk of infectious diseases.

## Methods

The following analysis was generated in international research (Australian Research Council Discovery Project DP1101081) focusing on the responses of members of the general public to the events of 2009 alongside interviews with researchers, clinicians and policy-makers [27,28] and analyses of the public policy texts on pandemic influenza control [29]. This research has examined general public data in light of sociological and psychological perspectives on responses to pandemic influenza [23,30-33]. The present paper synthesises and builds on the research undertaken on the general public, in particular, and introduces new data analysis to address the public health challenge of effective communication and engagement with members of the general public.

Interview and focus group participants were recruited through community sampling in Melbourne, Sydney and Glasgow. Generating data in Australia and Scotland addressed the international dimension of pandemic influenza and the events of 2009. Australia was closely observed by other nations as early stages of the global pandemic in 2009 coincided with the southern hemisphere influenza season. The pandemic quickly affected Melbourne, which reported a high and early peak of known infections [34,35]. The city, for a time was known as the ‘flu capital of the world.’ The first confirmed cases in the UK were in Scotland among passengers on a flight from Mexico to Glasgow [36]. The UK and Australia reported 457 [8] and 191 [37] deaths, respectively, associated with the 2009 H1N1 pandemic. Our analysis of interview and focus group texts reveals more convergence than difference between Melbourne, Sydney and Glasgow, perhaps because the pandemic was managed in those cities by public health professionals who were members of a global pandemic response network.

The research aimed to identify how members of the general public respond to pandemic influenza so that public health communications can be designed to engage with how its audiences respond to risk messages and how they enact hygiene, social isolation and related measures. Four purposive criteria were used to select respondents in each city: women who were pregnant during 2009 (or with a new baby); older members of the community (71 years of age and older); people with compromised immune systems and or respiratory illness such as asthma; and people who self-identified as being ‘healthy’ (e.g., no disclosed health problems) and who did not belong to one of the former categories. In addition, selection of participants was conducted to ensure: a balance of male and female participants and a range of ages from 18 years upwards. Drawing on interviews and focus groups ensured depth and breadth. Interviews explored in-depth discussion of personal experiences of living through the H1N1 pandemic, seasonal influenza and related concerns. Focus groups examined social norms concerning precautionary behaviours regarding pandemic influenza.

Between April 2011 and May 2012, 116 people participated in the research (see Table 1) in 57 interviews and ten focus groups (with 59 participants). Interviews included people from the purposive criteria (pregnant = 14; 71+ = 3; HIV/respiratory illness = 17; healthy = 23); a gender mix (women = 34; men = 23), and; an age range of 18 to 71+ years. Focus groups included people from the 71+ group (10); HIV/respiratory illness (37) and the healthy group (22); a gender mix (women = 36; men = 23), and; an age range of 18 to 71+ years. This pattern of participation reflects the challenges of recruiting women who were pregnant in 2009, the very elderly and men. Seven respondents reported having been diagnosed with H1N1; none through a laboratory-confirmed test (a reflection of our community sampling). A further eleven interviewees reported that a relative, friend or other social contact had been diagnosed, clinically. It needs to be acknowledged, however, that, as influenza is not ordinarily diagnosed with a laboratory confirmed test [38], public health professionals and members of the general public identify and manage the infection on the basis of symptoms. Indeed, respondents noted difficulty determining whether they had had influenza (H1N1 or another strain) because of variability in symptoms. Taken together, these figures demonstrate that a diverse sample was recruited which enabled data collection from members of the general public variously impacted by the 2009 H1N1 pandemic. The methods were approved by the Monash University Ethics Committee and participants provided written informed consent.

**Table 1:** **Sample by purposive selection criteria and city**

**City**	**Pregnant/ new baby in 2009**	**71 years of age and older**	**Immune compromised/ respiratory illness**	**Healthy**	**Total**
Sydney	3	6	18	26	53
Melbourne	6	4	10	17	37
Glasgow	6	0	16	4	26
Total	15	10	44	47	116

Participants were asked to speak about their experiences with influenza and the public health response to the 2009 pandemic. Topics for discussion included: health background (including pre-existing medical conditions, other infectious diseases, influenza vaccination); influenza experiences (including knowledge of pandemic influenza, sources of knowledge, experiences with the 2009 pandemic and seasonal influenza, prevention of infection, caring for self and/or someone else with infection); public communications (including broadcast and electronic media, public health advice, advice from GPs, workplace and schools). Verbatim transcripts of interviews and focus groups were analysed using an inductive, theory-building method. All transcripts were open coded to generate themes for analysis. Interpretive memoranda were generated which explained each theme and how it connected with existing perspectives on the general public response to pandemic influenza. The research team reviewed these themes and memoranda to ensure that the themes were understood and that they could withstand refutation. This discussion also provided the basis for an agreed coding scheme that was used to re-code all data. Key themes were identified for subsequent, in-depth written analysis in the form of technical reports and draft manuscripts. Our approach to coding, memo writing and in-depth analysis sustains a dialogue between theory (pre-existing categories derived from social science theory and the relevant literature) and data (inductively-derived themes). This approach avoids the traps of overly data- or theory-driven analysis and ensures that the research has relevance to the field. This paper, therefore, is based on in-depth, nuanced analysis of interview and focus group texts that offers new perspectives and propositions, which provide the basis for interrogating prevailing assumptions regarding the general public response to pandemic influenza. This approach is consistent with social inquiry of the highest standard [39].

## Results

The assumed complacency and resistance on the part of members of the general public was not in evidence in the narratives provided by our research participants. Other factors, centred around health individualism and contextual factors such as gender and biomedical situation do appear to influence how people respond to the threat of pandemic influenza. In what follows, we focus on themes that establish and complicate the role of health individualism and its effects in the responses of members of the general public to pandemic influenza.

### Self and other in responses to pandemic influenza

The interviews and focus groups revealed a tension to do with self and other in relation to the threat of pandemic influenza. As we have discussed elsewhere, respondents endorsed the pandemic control measures advocated by public health authorities [23]. They agreed that hygiene control measures (coughing and sneezing etiquette) and social distancing were valuable. This endorsement held in Australia and Scotland. Characteristically, however, respondents did not believe that pandemic influenza could be prevented in the long run. They believed that the influenza virus was easy to catch and that hygiene measures and social isolation were difficult given that social interaction was needed to sustain work, schooling, the family and daily life. For this reason, respondents focused on strengthening their immunity through, for example, taking vitamins and eating healthy food:

I think if you’re healthy, keep up your vitamins and eat the right foods, drink healthily, eat healthily and live healthily. Exercise. You’ve got to do all those things. (Heather, 71+, Melbourne)

This immunity boosting was seen as a prudent defence against the seemingly inevitable moment of exposure and a means of coping with infection when and if it occurred. Importantly, this focus on one’s body and immunity in the face of seemingly inevitable infection accentuated health individualism, encouraging members of the general public to focus on their body’s abilities to resist and cope with infection. There was evidence that immune boosting has the status of a social norm as those who were seen to succumb to infection were sometimes judged as failing to adequately care for themselves, even though it was admitted that the virus was easy to catch. To some extent individualism is an asset for public health interventions that seek behaviour change at the individual level. However, an individualistic approach to pandemic risk may obscure factors that the individual cannot control and, as indicated by the judgement of those who acquired infection, health individualism may be moralising.

Health individualism was not the only factor influencing how members of the general public perceived risk for pandemic influenza and took action. Respondents who had responsibilities for others (e.g., pregnant women, people in couples or caring for people with health problems, families with children) or who saw themselves as vulnerable to influenza (e.g., respiratory illness, immune disorders) focused on social units such as the couple, family and colleagues at work:

Well given that the flu broke out at XXXX Street Primary School and my son was three and he was at XXXX Street Childcare, I pulled him out. So when my husband picked him up that day I was at work. I said, ‘Take him home. Give him a bath. Wash his clothes.’ Yeah. I stopped sending him and I was one week off my maternity leave so I stopped work a week early … I didn’t go to the supermarket, didn’t really mix. (Gill, pregnant, Melbourne, 31 – 40 years)

It appears, then, that both health individualism and relationships with important others influence what people do. In this regard, social proximity appears to be important, that is, those others who are close to oneself in terms of social and emotional ties and living situation are factored into health precautions. This social proximity also showed up in the ways in which respondents saw geographical distance and low population density as protective. Those respondents living further away from the populous ‘epicentres’ of infection – central Melbourne, for example – believed that they were less likely to encounter someone with the virus.

### ‘We’re familiar with chest infections’

One important way in which this tension between responsibility to oneself and to others came to light in interviews and focus groups related to differences between the responses of those with pre-existing conditions and those who identified as ‘healthy.’ Those who faced increased risk of serious disease focused on their relationships with others – including strangers they might encounter in public spaces – largely in an effort to protect themselves. Those with no vulnerabilities showed themselves to be archetypally focused on their individual health. For example, people with severe respiratory illness reported that engagement with the risks of influenza was a ‘well trodden path’ for them:

As lung patients, we’re, we’re familiar with chest infections and, as Joy says, we could, we could have a flu and not know it. And the GP checks us over. And the only way that I know that they’ll know whether it’s a chest infection or flu, or pneumonia, is for an x-ray. (Arthur, Lung disease, Melbourne, 71+ years)

People with pre-existing lung conditions, then, were commonly hyper-vigilant during the 2009 pandemic and their accounts were peppered with examples of how social interaction was imbued with risk for them and also some resentment that the healthy majority seemed to not understand the significant threat that influenza infection might pose to their health [33]. People with immune disorders in our sample – primarily HIV – understood they needed to be vigilant but saw influenza as a lower priority than their HIV infection and its effective management. Older respondents (71+) conveyed judicious vigilance tempered with an unwillingness to be seen to overreact. Important in these accounts was awareness of the vectors of transmission and that one’s health was to some extent dependent on those with whom one interacted. In contrast, the healthy majority of our respondents saw pandemic influenza as a personal, though distant, health threat. They saw themselves ‘at risk’ and possibly as ‘a risk’ to close family, but not as ‘a risk’ to unknown others (e.g. a person sitting beside them on public transport). This focus on the ‘at risk’ self to the exclusion of the self as ‘a risk’ to others underlines how health individualism manifests in the responses of the ‘healthy’ majority of the general public.

### ‘Just ignore it and push through’

This focus of the healthy on their own health risks (at the expense of others) surfaced in narrative on expectations of recovery from influenza:

Like you sort of just, you think, maybe you just think influenza as a common cold sort of thing. And it’s like, ‘It’ll pass. I might go to the doctor’s and get some, something to help me get through it,’ or something. But yeah, I don’t know … It’s just like, ‘Just ignore it and push through.’ (Chris, healthy, Melbourne, 18 – 30 years)

This interview participant shows how a healthy individual engages with pandemic influenza as a commonplace and personal risk, in contrast to those with pre-existing conditions who have to take pandemic, and even seasonal, influenza seriously. This expectation that one can ‘push through’ reinforces the previous theme noted with regard to the focus on the capacity of one’s body to deal with infection. It is also an orientation to influenza risk that sets the scene for individuals to determine that infection is a risk worth taking since recovery is likely. Also, recovery expectations synergise with the belief that infection is difficult to avoid in the long run. This means that people may assume that, while non-pharmaceutical strategies of pandemic control are sensible, their limited utility is set against the likelihood of recovery. This nexus of risk calculation helps explain why segments of populations appear to be complacent in surveys, as noted above. They may in fact be making multi-layered risk assessments of the likelihood of infection, their health status and expectations of recovery.

### Gendering of influenza care and ‘man flu’

Another important provision on health individualism was the gendered meanings of one’s response to infection. Particularly in domestic settings, the management of respiratory illness was largely feminised. Women provided elaborate accounts of managing the respiratory infections of family members while men did not. Importantly, the pejorative term ‘man flu’ was used to denote the over-inflation of mild symptoms to gain sympathy and respite from normal activities, with connotations of questionable masculinity:

It’s always a little difficult to tell when you’re moving from, sort of, a cold through the man flu to proper influenza. (Vincent, healthy, Sydney, 41 – 50 years)

These findings imply that responses to pandemic influenza in real world settings are – as with other health problems – associated with gender roles which shape behaviour, for example, women may be expected to perform infection control and symptom management, while men are expected to not show their symptoms and ‘soldier on’ or face accusations of ‘man flu.’ The uniform implementation of social distancing and other protective measures may therefore be compromised.

### ‘Everything is about the baby’

Accentuating the role of gender in response to messages concerning pandemic influenza, pregnant women found themselves thrust into a position of particular risk during the 2009/10 pandemic, at a time when they were already taking responsibility for the well-being of their unborn child. In particular, the prospect of vaccination elicited varied, often emotion-laden, responses:

Well, (sigh) when you’re pregnant everything is about the baby … You just want to try and make your baby as healthy as possible and you want to try and keep your baby safe. (Rebecca, pregnant in 2009, Glasgow, 31 – 40 years)

The imperatives of good motherhood and responsibility for their unborn children placed these women into the emotionally-charged position of having to make decisions regarding virus protection in circumstances of intense uncertainty [32]. Some distress was apparent among the pregnant women respondents, but also great resilience and active use of public policy information to protect themselves and their babies.

### Vaccination: ‘That’s a bit of a Catch-22’

As Rebecca’s account, above, indicates, health individualism in tension with responsibilities to others, gender and one’s life situation played out in engagements with vaccination. Though recollection was variable, 64 respondents in the present research (55%) reported that they had had an influenza vaccination at some point in their lifetime and there was no evidence of ‘in principle’ resistance to vaccination. This is a notable finding given that participants were sought in community settings – where those with anti-vaccine views are thought to be located – and in light of commentary that members of the general public are resistant to the science and technology used to manage global threats. Indeed, endorsement of public health measures and attempted compliance characterised the respondents’ accounts, with the provisos on the practical value of non-pharmaceutical strategies of infection control and management, as already discussed. But, taking on vaccination was not always straightforward:

I saw in the press releases about the vaccine and I remember ringing the clinic and they said, ‘Well if we were to give it to you, you’d have to come to the hospital and that’s gonna put you at risk of getting exposed to it so we’d rather you not come in for the, for the vaccine.’ And I was thinking, ‘Well that’s a bit of a catch-22.’ (Cindy, Lung disease, Sydney, 18 – 30 years)

For someone with a specific vulnerability, then, accessing vaccination presented a problem of relative risks – in this case, of reducing the threat of infection through vaccination combined with the risk of exposure in a clinical setting – underscoring the importance of one’s life circumstances in the management of the threat of influenza. Respondents also noted that the good reputation of vaccination waxed and waned, in this case, in the context of the 2009/2010 pandemic:

One minute everybody’s going, ‘I wanna vaccination,’ and the next minute everyone’s too paranoid. (Jan, Lung disease/pregnant in 2009, Melbourne, 31–40 years)

Importantly, though, vaccination, like non-pharmaceutical infection control, was mostly discussed as a personal strategy of health protection. Apart from those with pre-existing vulnerabilities, vaccination was not readily understood as a method for protecting others and therefore society. This individualistic focus on one’s own health implies that efforts to promote ‘herd immunity’ may not accord with perceptions and behaviours of the healthy majority.

## Discussion

The findings question the prevailing view that the general public resists risk communication with regard to pandemic influenza. Nor do the related ideas of complacency and fatigue seem relevant. More salient was multi-layered risk management informed by health individualism and to some extent tempered by interpersonal responsibilities, one’s personal circumstances, gender, expectations of recovery, and prior experiences with influenza. As others using qualitative methods have also suggested [19], respondents did not reject what was done by governments in 2009. They show interest in pandemic influenza, though their mode of engagement with it varied. They indicated that they wished to be informed but reserved the right to interpret and apply advice according to their own situation. Public health guidance on hygiene and social isolation was endorsed, though its utility was largely found to have practical, long-term limitations given that social interaction was fundamental to daily life and the transmission of the virus.

Resistance and the related notions of complacency and fatigue, then, appear to have limited value for explaining how members of the general public respond to pandemics. Part of the reason for this inapt attribution of research results to public resistance concerns research approach. Forced choice surveys produce measures of hypothesised variables thought to influence behaviour. In-depth interviews and focus groups yield a different picture, where general public perceptions of the dangers of pandemics are placed in the context of what appears to be endorsement of the efforts of public health, tempered with awareness of the practical difficulties of managing influenza on a local basis. Personal experience narratives reveal members of the general public to be engaged and willing to apply guidance in real world settings, though also aware of limits on what might be possible in time of pandemic.

Going beyond the idea of resistance, our analysis offers an alternative framing of how members of the general public respond to pandemic influenza. Health individualism complicated by life circumstances (family life, health status) and the gendering of the meanings and practices surrounding the experience of influenza and how to deal with it in real world settings, appear to be important. Risk communications are likely to benefit by addressing these influences on risk management behaviours. In particular, emphasising individual responsibility in risk communication may amplify divisions between people with different biomedical vulnerabilities and encourage those who consider themselves healthy to think of themselves as ‘at risk’ but not ‘a risk’ to others. This is a major hurdle for public health, particularly when hygiene, social isolation and vaccination are likely to become more important methods for controlling the spread of re-emerging infectious diseases. The pejorative, gendered meanings of influenza, of which ‘man flu’ stands as exemplary, point towards the deeply inscribed gendering of responses to infectious diseases. The role of gender in social aspects of health care is no surprise, but fully-fledged gender analysis is yet to be acknowledged in the public health address to the general population with regard to pandemics. In particular, messages to enact hygiene and social isolation are likely to accentuate already feminised health care in the domestic sphere. Further, it is not simply that women are burdened with the labour of influenza care and men not. If men do find themselves unwell they risk accusations of ‘man flu’ and may therefore avoid making themselves available for health care interventions, a dynamic which keeps men out of the GP clinic in general [40]. As recent reviews have indicated [7,16], the influences of social factors on responses to pandemics need to be foregrounded in the social research agenda for better public health. Our research indicates that health individualism and gender need to be part of this new research agenda.

Our findings also point to several further, specific, challenges for risk communication: ideas of proximity to risk; expectations of recovery, and; vaccination. Proximity appears to be a blind spot in risk communications. Public health messages of emergency are filtered by perceptions of proximity to threat, consistent with psychological theory [41] and cultural constructs where the source of contagion is placed at a distance from self [42]. We found that these ideas of proximity did surface in the narratives of members of the general public. Yet, we know that, for example, within six weeks of the infection being detected in Australia, people in remote communities in Australia were found to be infected [43]. Risk communication needs to attend to these ideas of distance from risk and the related underestimation of the speed with which the influenza virus can travel in a hyper-connected world.

Expectations of recovery from influenza also appear to dominate narratives. As others have argued [44], healthy respondents recognised influenza infection as severe – requiring bed and rest – but thought that they would eventually recover. This finding implies that members of the general public may interpret infection as a risk worth taking, that is, that they can cope with infection if prevention fails them, due to their own choices or otherwise. Members of the general public appear to be actively engaged with manifold risks that they juggle and prioritise in real world settings.

Our findings also suggest that taking up vaccination is not a simple matter, even among those who endorse the use of the biotechnology. Survey findings have found that approximately 42% of Australians are concerned about general vaccine safety [14] and that Australian [45] and worldwide [46] rates of H1N1 vaccination have been found to be insufficient, prompting concerns that the ‘anti-vaccine lobby’ and other detractors are influencing use of this biotechnology. As noted, a slight majority of our respondents reported that they had been vaccinated in their lifetime and none spoke of vaccination as dangerous, though, of course, some may have held these views and not revealed them or opted out of our community-based recruitment strategies. Our research, however, points to more immediate and practical considerations that shape how and when people vaccinate, including considerations of relative risk and whether or not a new vaccine should be used in pregnancy. Attending to these more immediate concerns may be beneficial for public health, though we acknowledge that public perception of vaccine technologies is also an important public health agenda.

The analysis presented is retrospective as the interviews and focus groups were conducted after the end of the pandemic on 10 August 2010 [47], and therefore when it was known that the mortality rate had at first been overestimated [48]. Importantly, too, the respondents were volunteers selected according to purposive criteria, implying that the sample is not representative and that generalisations to populations are not strictly tenable. What the analysis offers, however, is the opportunity to drill down into how people make sense of pandemic influenza, therefore providing the basis for building theory on how members of the general public, think, feel and act in the contemporary era of efforts to manage global health threats. The perspectives identified here help situate what we know in social context and alert public policy to some dilemmas and alternative explanations of why members of the general public do what they do.

## Conclusions

For public health to shape the actions people take prior to and during a pandemic, we need to understand and engage with the perspectives of those acting. Viewed from the outside, the behaviour of the general public has been cast as resistant. However, viewed from the perspective of ordinary people involved in anticipating and responding to infection, it is clear that public health has engaged its publics. This engagement is frequently informed by individualistic ways of assessing and responding to risk, social norms (e.g. gender roles), knowledge of the clinical uncertainties of influenza infection, and reasoned thinking about the limits of preventing influenza transmission. The current challenge for pandemic influenza preparedness and response is not so much to address public disinterest, but to acknowledge and engage with members of the general publics’ experiences of influenza and how they make sense of, and act on, pandemics in real world settings.
